# Spontaneous Ca^++ ^oscillations in astrocytes initiate high-frequency oscillations in model hippocampal network

**DOI:** 10.1186/1471-2202-14-S1-P293

**Published:** 2013-07-08

**Authors:** Vivek Nagaraj, Theoden Netoff

**Affiliations:** 1 Graduate Program in Neuroscience, University of Minnesota, Minneapolis, MN 55454, USA; 2 Biomedical Engineering, University of Minnesota, Minneapolis, MN 55454, USA

## 

**Figure 1:**
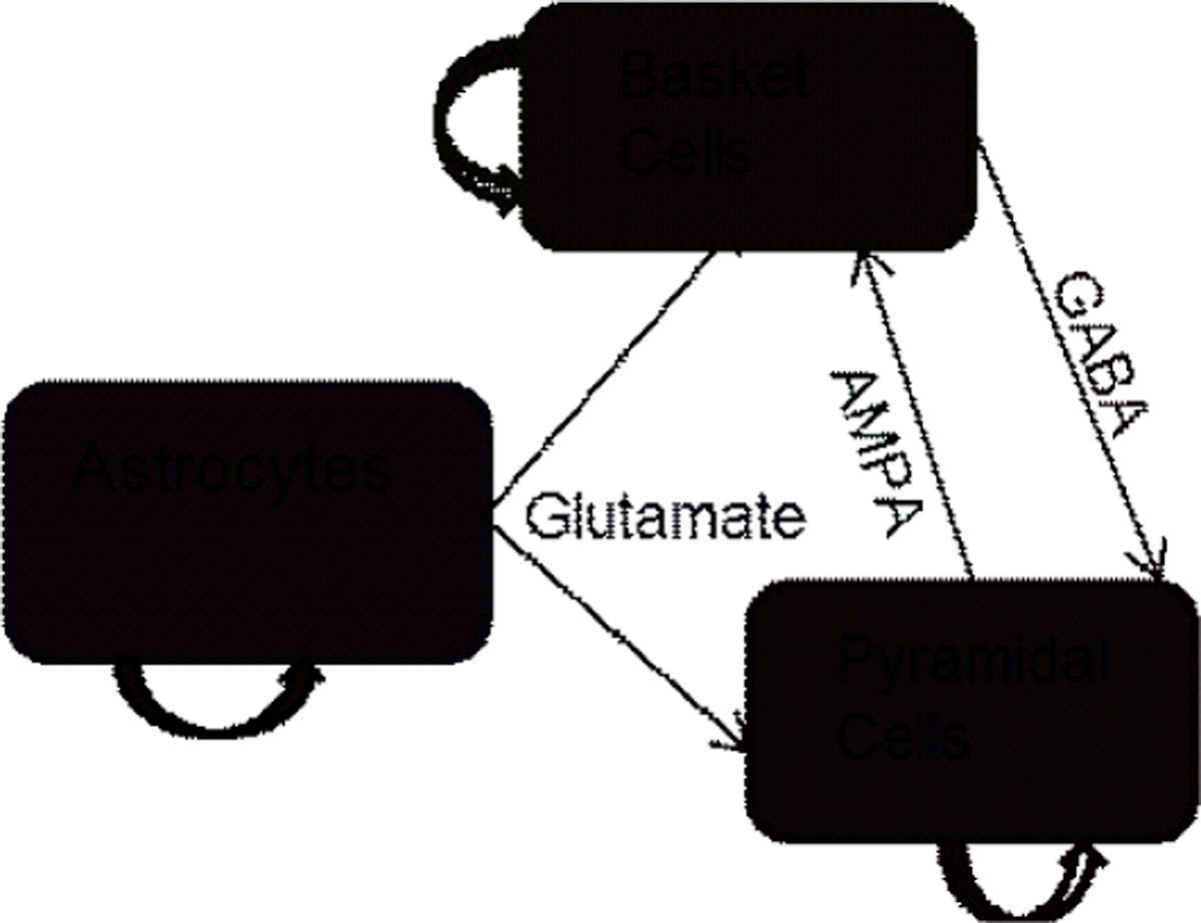
**A reduced three cell hippocampal circuit**. The curved arrows correspond to gap-junction coupling within the population. Gliotransmitter effects are modeled as inward current into the neuronal population following elevation in calcium within astrocytes. Basket cells and pyramidal cells are coupled through chemical synapses. All cell populations are continuously perturbed by noise.

Tonic-Clonic (TC) seizures typically follow episodes of high frequency oscillations (HFOs). The dynamics of these HFOs have been studied in various types of animal and computational seizure models. Hippocampal slice experiments have shown high frequency activity can be decoupled through the use of gap-junction blockers[1]. This has generally been attributed to gap-junctions between neurons, however we hypothesize that this may be caused by blocking gap-junctions been astrocytes. To test this hypothesis we constructed a model network consisting of three distinct cell populations (Figure 1). Spontaneous calcium oscillations through astrocytes cause a release of glutamate driving the neuronal population to fire with high frequency (100-140 Hz) oscillations. By decreasing gap-junction conductance within the pyramidal and basket cell populations no noticeable difference in high frequency power was seen in the time-frequency analysis, suggesting that HFOs may not require electrical coupling between neurons. Next, to test the role of calcium waves in generation of high frequency activity we decreased gap-junction conductance between all astrocytes. This resulted in a decrease in HFO power between 100-140 Hz. These results of the simulations suggest that spontaneous calcium waves through astrocytic gap junctions may induce high-frequency activity in reduced hippocampal pyramidal populations. Pathology studies of patients with mesial Temporal Lobe Epilepsy have shown an increase in astrocytic connexin43 expression, the main component in astrocyte gap-junctions[2]. The results of this experiment open a new opportunity in targeted drug therapies for epileptics, one that specifically targets astrocyte gap-junctions.
